# Di-*tert*-butyl *N*-{[1-(pyridin-4-yl)-1*H*-1,2,3-triazol-4-yl]methyl}iminodiacetate

**DOI:** 10.1107/S1600536812042596

**Published:** 2012-10-20

**Authors:** Alison François, Louise Marty, Claude Picard, Sonia Mallet-Ladeira, Eric Benoist

**Affiliations:** a LSPCMIB, UMR-CNRS 5068, Université de Toulouse, 118 route de Narbonne, F-31062 Toulouse cedex 9; b Université de Toulouse, UPS and CNRS, Institut de Chimie de Toulouse, FR2599, 118 route de Narbonne, F-31062 Toulouse cedex 9, France

## Abstract

In the title compound, C_20_H_29_N_5_O_4_, the pyridine ring makes a dihedral angle of 10.41 (16)° with the triazole ring, which exhibits an azo-like character. In the crystal, mol­ecules are linked by C—H⋯O and C—H⋯N hydrogen bonds, and C—H⋯π inter­actions involving a methyl group and the pyridine ring of a neighbouring mol­ecule, leading to the formation of a three-dimensional network.

## Related literature

For 4-pyridyl-1,2,3-triazoles as building blocks in the synthesis of chelating agents for biomedical applications, see: Bonnet *et al.* (2012 ▶); Pellegatti *et al.* (2008 ▶). For the crystal structures of structural isomers such as 2-pyridyl-1,2,3-triazoles, see: Obata *et al.* (2008 ▶); Schweinfurth *et al.* (2008 ▶); Boulay *et al.* (2010 ▶); Seridi *et al.* (2011 ▶); Crowley *et al.* (2010 ▶); Kilpin *et al.* (2011 ▶).
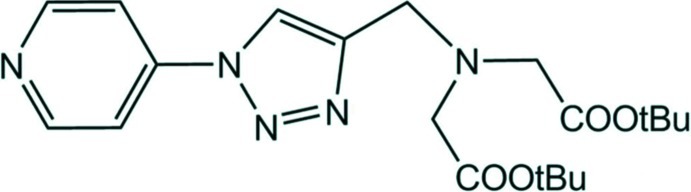


## Experimental

### 

#### Crystal data

C_20_H_29_N_5_O_4_
*M* *_r_* = 403.48
Monoclinic, 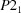

*a* = 9.1568 (8) Å
*b* = 11.4452 (10) Å
*c* = 11.4928 (11) Å
β = 110.840 (4)°
*V* = 1125.66 (18) Å^3^
*Z* = 2
Mo *K*α radiation
μ = 0.09 mm^−1^
*T* = 193 K
0.2 × 0.1 × 0.04 mm

#### Data collection

Bruker Kappa APEXII Quazar diffractometer
Absorption correction: multi-scan (*SADABS*; Bruker, 2008 ▶) *T*_min_ = 0.989, *T*_max_ = 0.997
11329 measured reflections
3530 independent reflections
1947 reflections with *I* > 2σ(*I*)
*R*_int_ = 0.077

#### Refinement

*R*[*F* ^2^ > 2σ(*F* ^2^)] = 0.059
*wR*(*F* ^2^) = 0.122
*S* = 1.00
3530 reflections
268 parameters
1 restraint
H-atom parameters constrained
Δρ_max_ = 0.18 e Å^−3^
Δρ_min_ = −0.18 e Å^−3^

### 

Data collection: *APEX2* (Bruker, 2008 ▶); cell refinement: *APEX2* and *SAINT* (Bruker, 2008 ▶); data reduction: *SAINT*; program(s) used to solve structure: *SHELXS97* (Sheldrick, 2008 ▶); program(s) used to refine structure: *SHELXL97* (Sheldrick, 2008 ▶); molecular graphics: *ORTEP-3 for Windows* (Farrugia, 2012 ▶); software used to prepare material for publication: *WinGX* publication routines (Farrugia, 2012 ▶) and *publCIF* (Westrip, 2010 ▶).

## Supplementary Material

Crystal structure: contains datablock(s) global, I. DOI: 10.1107/S1600536812042596/su2510sup1.cif

Structure factors: contains datablock(s) I. DOI: 10.1107/S1600536812042596/su2510Isup2.hkl

Supplementary material file. DOI: 10.1107/S1600536812042596/su2510Isup3.cml

Additional supplementary materials:  crystallographic information; 3D view; checkCIF report

## Figures and Tables

**Table 1:** Hydrogen-bond geometry (Å, °) *Cg*1 is the centroid of the N1/C1–C5 ring.

*D*—H⋯*A*	*D*—H	H⋯*A*	*D*⋯*A*	*D*—H⋯*A*
C2—H2⋯O1^i^	0.95	2.54	3.443 (4)	160
C6—H6⋯N1^ii^	0.95	2.31	3.252 (4)	173
C9—H9*B*⋯N3^iii^	0.99	2.50	3.449 (4)	160
C18—H18*A*⋯*Cg*1^iv^	0.98	2.91	3.864 (4)	166

Symmetry codes: (i) 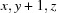; (ii) 
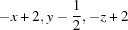
; (iii) 
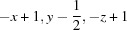
; (iv) 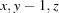.

**Fig. 1.**
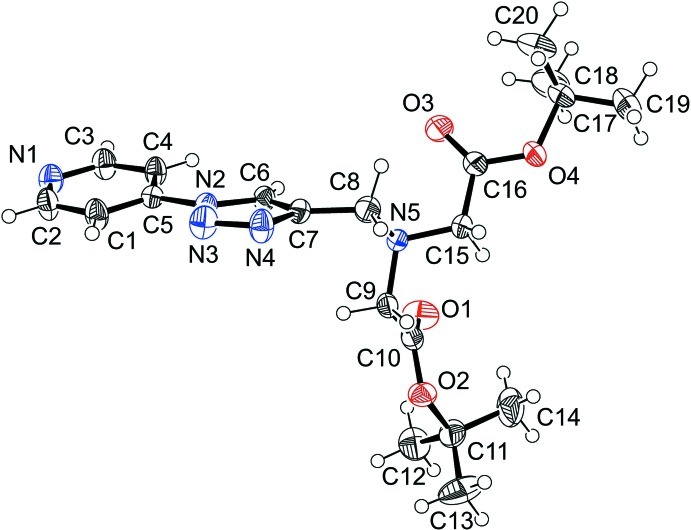
The molecular structure of the title compound with the atom numbering. The displacement ellipsoids are drawn at the 50% probability level.

**Fig. 2.**
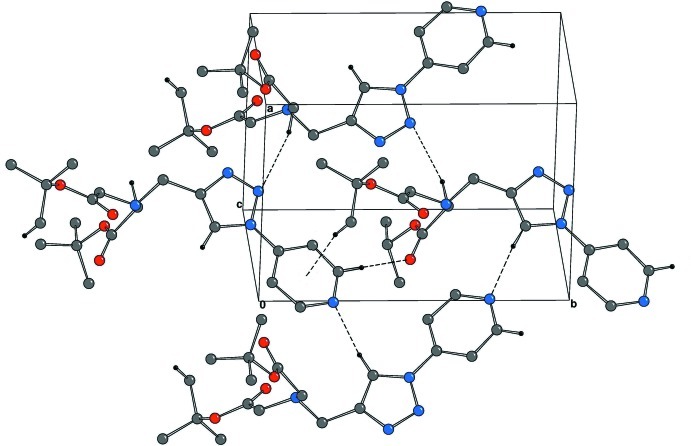
A view of the crystal packing of the title compound, showing the C-H···O and C-H···N hydrogen bonds and the C—H··· π interactions (dashed lines). H atoms not involved in these interactions have been omitted for clarity.

## supplementary crystallographic information

### Comment

If 1,2,3-Triazoles are well known for their biological properties, particular attention has been recently devoted to the development of 2-pyridyl-1,2,3-triazole derivatives (or pyta) as alternative ligands to 2,2'-bipyridines. This interest is explained by the easy preparation of such ligands using a click chemistry strategy (Obata *et al.*, 2008; Schweinfurth *et al.*, 2008), and the use of pyta derivatives as efficient chelator systems for Tc(CO)_3_^+^ or Re(CO)_3_^+^ organometallic cores (Boulay *et al.*, 2010; Seridi *et al.*, 2011). Recently, structural pyta isomers like 4-pyridyl-1,2,3-triazole have been described as building blocks in the synthesis of chelating agents for biomedical applications (Bonnet *et al.*, 2012; Pellegatti *et al.*, 2008). In this paper, we report on the first X-ray structure analysis of a 4-pyridyl-1,2,3-triazole derivative.

The title molecule, Fig. 1, can be considered as a ditopic ligand with two distinct transition metal complexing sites, the iminodiacetate (IDA) pincer and the 4-pyridine moiety. Bond lengths and angles are within normal ranges, and comparable with values found for structural isomers, such as 2-pyridyl-1,2,3-triazole derivatives (Obata *et al.* 2008; Schweinfurth *et al.*, 2008; Seridi *et al.*, 2011; Boulay *et al.*, 2010). As is often observed in these ligand systems, the pyridyl and triazole units are coplanar (Crowley *et al.*, 2010; Kilpin *et al.*, 2011). Unarguably, the structure exhibits a practically planar geometry with slight deviation of the pyridyl moiety, which makes a dihedral angle of 10.41 (16)° with the mean plane of the triazole ring. As expected, the N3–N4 distance of the 1,2,3-triazole at 1.308 (4) Å is shorter than the N4–C7 and N2–N3 bonds, 1.358 (4) and 1.356 (3) Å respectively, confirming the azo character of the triazolyl entity.

In the crystal, molecules are linked by C–H···O and C–H···N hydrogen bonds (Table 1 and Fig. 2). It is noteworthy that a C–H···π interaction between of the hydrogen H18A of one methyl group and the π cloud of the pyridine ring was also observed, this interaction participates in the cohesion of the crystal.

### Experimental

Freshly prepared 4-azidopyridine (0.4 g, 3.3 mmol), 3-[bis(*tert*-butoxycarbonylmeth-yl) amino]-prop-1-yne (0.94 g, 3.3 mmol), copper(II) acetate monohydrate (130 mg, 0.66 mmol) and sodium ascorbate (260 mg, 1.32 mmol) were mixed in acetonitrile (5 ml) and stirred overnight at 303 K. The resulting brown solution was cooled then diluted with chloroform (10 ml) and washed twice with saturated Na_2_edta solution (2x15 ml). The aqueous solutions were extracted with chloroform (3x7 ml). The organic extracts were combined, dried over Na_2_SO_4_ and the solvent was taken off under reduce pressure. The crude product was purified by column chromatography on neutral alumina (eluent: CH_2_Cl_2_) to give 1.03 g of the title compound [Yield: 77%]. Analysis calculated for C_20_H_29_N_5_O_4_: C 59.54, H 7.24, N 17.36%; found: C 59.24, H 7.32, N 17.44%. Plate-like colourless crystals, suitable for X-ray diffraction analysis, were obtained by slow evaporation of a methanol-dichloromethane (1:1 / v:v) solution. Further spectroscopic data for the title compound are available in the archived CIF.

### Refinement

All the H atoms were included in calculated positions and treated as riding atoms: C—H = 0.95 Å (aromatic), 0.99 Å (methylene) and 0.98 Å (methyl) with *U*_iso_(H) = 1.2*U*_eq_(aromatic, methylene) or *U*_iso_(H) = 1.5*U*_eq_(methyl). In the final cycles of refinement, in the absence of significant anomalous scattering effects, 2547 Friedel pairs were merged and \Df " set to zero.

### Crystal data

**Table:**

C_20_H_29_N_5_O_4_	*F*(000) = 432
*M**_r_* = 403.48	*D*_x_ = 1.19 Mg m^−^^3^
Monoclinic, *P*2_1_	Mo *K*α radiation, λ = 0.71073 Å
Hall symbol: P 2yb	Cell parameters from 1261 reflections
*a* = 9.1568 (8) Å	θ = 2.4–21.3°
*b* = 11.4452 (10) Å	µ = 0.09 mm^−^^1^
*c* = 11.4928 (11) Å	*T* = 193 K
β = 110.840 (4)°	Plate, colourless
*V* = 1125.66 (18) Å^3^	0.2 × 0.1 × 0.04 mm
*Z* = 2	

### Data collection

**Table:**

Bruker Kappa APEXII Quazar diffractometer	3530 independent reflections
Radiation source: microfocus sealed tube	1947 reflections with *I* > 2σ(*I*)
Multilayer optics monochromator	*R*_int_ = 0.077
phi and ω scans	θ_max_ = 30.6°, θ_min_ = 3.0°
Absorption correction: multi-scan (*SADABS*; Bruker, 2008)	*h* = −13→11
*T*_min_ = 0.989, *T*_max_ = 0.997	*k* = −14→16
11329 measured reflections	*l* = −16→16

### Refinement

**Table:**

Refinement on *F*^2^	Primary atom site location: structure-invariant direct methods
Least-squares matrix: full	Secondary atom site location: difference Fourier map
*R*[*F*^2^ > 2σ(*F*^2^)] = 0.059	Hydrogen site location: inferred from neighbouring sites
*wR*(*F*^2^) = 0.122	H-atom parameters constrained
*S* = 1.00	*w* = 1/[σ^2^(*F*_o_^2^) + (0.0464*P*)^2^] where *P* = (*F*_o_^2^ + 2*F*_c_^2^)/3
3530 reflections	(Δ/σ)_max_ = 0.001
268 parameters	Δρ_max_ = 0.18 e Å^−^^3^
1 restraint	Δρ_min_ = −0.18 e Å^−^^3^

### Special details

**Table:**

Experimental. Spectroscopic data for the title compound: ^1^H NMR (300 MHz, CDCl_3_): δ/p.p.m. = 1.45 (s, 18H, CH_3_); 3.49 (s, 4H, CH_2_); 4.12 (s, 2H, CH_2_); 7.72 (m, 2H, CH_Ar_); 8.24 (s, 1H, CH_ta_); 8.75 (m, 2H, CH_Ar_); ^13^C NMR (75 MHz, CDCl_3_): δ/p.p.m. = 28.1 (9CH_3_); 49.0 (CH_2_); 55.5 (2CH_2_); 81.3 (2C_IV_); 113.6, 151.6 (4CH_Ar_); 120.7 (CH_ta_); 143.1, 147.5 (2C_IV_); 170.3 (2CO); IR (KBr): ν_C=O_ = 1735 cm^-1^; MS (DCI/NH_3_): m/*z* 404, [*M*^+^]; 426, [*M*+Na^+^]; 443, [*M*+K^+^].
Geometry. All e.s.d.'s (except the e.s.d. in the dihedral angle between two l.s. planes) are estimated using the full covariance matrix. The cell e.s.d.'s are taken into account individually in the estimation of e.s.d.'s in distances, angles and torsion angles; correlations between e.s.d.'s in cell parameters are only used when they are defined by crystal symmetry. An approximate (isotropic) treatment of cell e.s.d.'s is used for estimating e.s.d.'s involving l.s. planes.
Refinement. Refinement of *F*^2^ against ALL reflections. The weighted *R*-factor *wR* and goodness of fit *S* are based on *F*^2^, conventional *R*-factors *R* are based on *F*, with *F* set to zero for negative *F*^2^. The threshold expression of *F*^2^ > σ(*F*^2^) is used only for calculating *R*-factors(gt) *etc*. and is not relevant to the choice of reflections for refinement. *R*-factors based on *F*^2^ are statistically about twice as large as those based on *F*, and *R*- factors based on ALL data will be even larger.

### Fractional atomic coordinates and isotropic or equivalent isotropic displacement parameters (Å^2^)

**Table:**

	*x*	*y*	*z*	*U*_iso_*/*U*_eq_	
O2	0.8574 (2)	0.02696 (19)	0.4779 (2)	0.0380 (5)	
N1	1.0194 (3)	0.7418 (2)	0.9730 (2)	0.0392 (7)	
O3	0.6055 (3)	0.0266 (2)	0.8927 (2)	0.0521 (7)	
O4	0.5024 (3)	−0.13966 (19)	0.7905 (2)	0.0378 (6)	
N5	0.6572 (3)	0.1115 (2)	0.6817 (2)	0.0276 (6)	
N3	0.5993 (3)	0.5068 (2)	0.6577 (2)	0.0407 (7)	
N4	0.5251 (3)	0.4092 (3)	0.6151 (2)	0.0393 (7)	
C4	0.9274 (3)	0.5441 (3)	0.9510 (3)	0.0342 (8)	
H4	0.9346	0.4682	0.9859	0.041*	
C17	0.4539 (4)	−0.1904 (3)	0.8901 (3)	0.0442 (9)	
C6	0.7207 (4)	0.3635 (3)	0.7862 (3)	0.0293 (7)	
H6	0.7926	0.3216	0.8537	0.035*	
C7	0.5962 (3)	0.3197 (3)	0.6918 (3)	0.0278 (7)	
C10	0.8501 (4)	0.0438 (3)	0.5901 (3)	0.0323 (7)	
C9	0.7146 (3)	0.1245 (3)	0.5787 (3)	0.0290 (7)	
H9A	0.7481	0.2064	0.576	0.035*	
H9B	0.6281	0.1081	0.4993	0.035*	
N2	0.7201 (3)	0.4797 (2)	0.7634 (2)	0.0277 (6)	
C5	0.8218 (3)	0.5684 (3)	0.8340 (3)	0.0279 (7)	
C8	0.5345 (3)	0.1981 (3)	0.6690 (3)	0.0335 (8)	
H8A	0.4823	0.1794	0.7288	0.04*	
H8B	0.4551	0.1931	0.584	0.04*	
O1	0.9358 (3)	0.0025 (2)	0.6860 (2)	0.0511 (7)	
C16	0.5703 (4)	−0.0346 (3)	0.8019 (3)	0.0339 (7)	
C15	0.5930 (4)	−0.0054 (3)	0.6821 (3)	0.0299 (7)	
H15A	0.6644	−0.0636	0.6672	0.036*	
H15B	0.4912	−0.0113	0.6128	0.036*	
C2	0.9161 (4)	0.7613 (3)	0.8589 (3)	0.0408 (8)	
H2	0.9119	0.8378	0.8258	0.049*	
C3	1.0224 (4)	0.6337 (3)	1.0161 (3)	0.0393 (8)	
H3	1.0948	0.6169	1.097	0.047*	
C11	0.9656 (4)	−0.0587 (3)	0.4568 (3)	0.0440 (9)	
C14	0.9375 (5)	−0.1775 (3)	0.5026 (5)	0.0735 (14)	
H14A	0.9729	−0.177	0.5937	0.11*	
H14B	0.9957	−0.2369	0.4755	0.11*	
H14C	0.8256	−0.1957	0.4682	0.11*	
C19	0.3794 (6)	−0.3030 (4)	0.8319 (4)	0.0722 (13)	
H19A	0.2924	−0.2864	0.7542	0.108*	
H19B	0.34	−0.3444	0.8893	0.108*	
H19C	0.4569	−0.3516	0.814	0.108*	
C1	0.8158 (4)	0.6794 (3)	0.7858 (3)	0.0380 (8)	
H1	0.7448	0.6984	0.705	0.046*	
C12	1.1312 (4)	−0.0187 (4)	0.5169 (4)	0.0566 (11)	
H12A	1.1436	0.0581	0.484	0.085*	
H12B	1.2012	−0.0749	0.499	0.085*	
H12C	1.1573	−0.0133	0.6072	0.085*	
C18	0.5965 (5)	−0.2123 (4)	1.0045 (4)	0.0658 (12)	
H18A	0.6712	−0.2602	0.9821	0.099*	
H18B	0.5658	−0.2534	1.067	0.099*	
H18C	0.6451	−0.1375	1.0386	0.099*	
C20	0.3373 (5)	−0.1108 (4)	0.9149 (4)	0.0656 (12)	
H20A	0.3908	−0.0407	0.9584	0.098*	
H20B	0.2886	−0.1516	0.9667	0.098*	
H20C	0.2567	−0.0884	0.8357	0.098*	
C13	0.9166 (5)	−0.0569 (4)	0.3165 (4)	0.0776 (15)	
H13A	0.8057	−0.0775	0.279	0.116*	
H13B	0.9791	−0.1135	0.2904	0.116*	
H13C	0.933	0.0215	0.2892	0.116*	

### Atomic displacement parameters (Å^2^)

**Table:**

	*U*^11^	*U*^22^	*U*^33^	*U*^12^	*U*^13^	*U*^23^
O2	0.0374 (12)	0.0399 (14)	0.0433 (13)	0.0053 (11)	0.0224 (10)	0.0003 (11)
N1	0.0382 (15)	0.0331 (17)	0.0403 (16)	−0.0034 (14)	0.0067 (12)	−0.0002 (13)
O3	0.0732 (17)	0.0544 (16)	0.0345 (13)	−0.0248 (14)	0.0262 (12)	−0.0158 (12)
O4	0.0505 (13)	0.0322 (13)	0.0355 (12)	−0.0114 (11)	0.0213 (10)	0.0007 (10)
N5	0.0300 (13)	0.0248 (14)	0.0315 (13)	−0.0036 (12)	0.0151 (11)	−0.0047 (10)
N3	0.0432 (15)	0.0378 (17)	0.0291 (14)	0.0000 (14)	−0.0020 (12)	0.0014 (12)
N4	0.0437 (16)	0.0287 (16)	0.0354 (15)	−0.0019 (14)	0.0015 (13)	0.0022 (12)
C4	0.0378 (18)	0.0237 (17)	0.0341 (17)	0.0005 (15)	0.0044 (14)	0.0039 (14)
C17	0.058 (2)	0.043 (2)	0.043 (2)	−0.012 (2)	0.0311 (18)	0.0024 (16)
C6	0.0325 (16)	0.0212 (16)	0.0337 (17)	0.0046 (14)	0.0112 (13)	0.0041 (13)
C7	0.0296 (16)	0.0232 (17)	0.0307 (16)	0.0021 (14)	0.0107 (13)	−0.0003 (13)
C10	0.0341 (16)	0.0273 (18)	0.0374 (18)	−0.0035 (15)	0.0150 (14)	0.0013 (14)
C9	0.0305 (15)	0.0227 (17)	0.0354 (17)	−0.0017 (14)	0.0136 (13)	0.0010 (13)
N2	0.0302 (12)	0.0239 (15)	0.0259 (13)	0.0025 (12)	0.0061 (10)	0.0020 (11)
C5	0.0308 (15)	0.0259 (17)	0.0280 (15)	−0.0020 (14)	0.0118 (13)	−0.0014 (13)
C8	0.0284 (15)	0.034 (2)	0.0391 (18)	−0.0007 (15)	0.0129 (14)	−0.0032 (14)
O1	0.0452 (13)	0.0633 (18)	0.0441 (14)	0.0205 (14)	0.0151 (11)	0.0161 (13)
C16	0.0348 (17)	0.0346 (19)	0.0352 (18)	−0.0056 (15)	0.0160 (14)	−0.0026 (14)
C15	0.0347 (16)	0.0261 (17)	0.0297 (15)	−0.0065 (15)	0.0124 (13)	−0.0057 (13)
C2	0.051 (2)	0.0250 (18)	0.0391 (19)	−0.0054 (17)	0.0077 (16)	0.0046 (15)
C3	0.0398 (18)	0.033 (2)	0.0362 (19)	−0.0009 (16)	0.0027 (15)	0.0052 (15)
C11	0.041 (2)	0.037 (2)	0.063 (2)	0.0023 (17)	0.0302 (18)	−0.0075 (17)
C14	0.075 (3)	0.035 (2)	0.129 (4)	0.000 (2)	0.060 (3)	−0.011 (3)
C19	0.098 (3)	0.058 (3)	0.074 (3)	−0.034 (3)	0.047 (3)	0.001 (2)
C1	0.0471 (18)	0.0270 (19)	0.0316 (17)	−0.0006 (17)	0.0039 (14)	0.0083 (14)
C12	0.039 (2)	0.054 (3)	0.087 (3)	0.006 (2)	0.035 (2)	−0.001 (2)
C18	0.079 (3)	0.077 (3)	0.046 (2)	0.002 (3)	0.029 (2)	0.019 (2)
C20	0.067 (3)	0.082 (3)	0.067 (3)	−0.006 (3)	0.048 (2)	0.005 (2)
C13	0.074 (3)	0.106 (4)	0.065 (3)	0.016 (3)	0.041 (2)	−0.023 (3)

### Geometric parameters (Å, º)

**Table:**

O2—C10	1.328 (4)	C8—H8B	0.99
O2—C11	1.474 (4)	C16—C15	1.501 (4)
N1—C3	1.329 (4)	C15—H15A	0.99
N1—C2	1.335 (4)	C15—H15B	0.99
O3—C16	1.202 (4)	C2—C1	1.371 (4)
O4—C16	1.339 (4)	C2—H2	0.95
O4—C17	1.486 (4)	C3—H3	0.95
N5—C15	1.461 (4)	C11—C12	1.497 (5)
N5—C9	1.464 (4)	C11—C13	1.512 (5)
N5—C8	1.466 (4)	C11—C14	1.513 (5)
N3—N4	1.308 (4)	C14—H14A	0.98
N3—N2	1.356 (3)	C14—H14B	0.98
N4—C7	1.358 (4)	C14—H14C	0.98
C4—C5	1.377 (4)	C19—H19A	0.98
C4—C3	1.379 (4)	C19—H19B	0.98
C4—H4	0.95	C19—H19C	0.98
C17—C19	1.499 (5)	C1—H1	0.95
C17—C20	1.506 (5)	C12—H12A	0.98
C17—C18	1.508 (5)	C12—H12B	0.98
C6—N2	1.355 (4)	C12—H12C	0.98
C6—C7	1.359 (4)	C18—H18A	0.98
C6—H6	0.95	C18—H18B	0.98
C7—C8	1.489 (4)	C18—H18C	0.98
C10—O1	1.200 (4)	C20—H20A	0.98
C10—C9	1.514 (4)	C20—H20B	0.98
C9—H9A	0.99	C20—H20C	0.98
C9—H9B	0.99	C13—H13A	0.98
N2—C5	1.421 (4)	C13—H13B	0.98
C5—C1	1.380 (4)	C13—H13C	0.98
C8—H8A	0.99		
			
C10—O2—C11	121.7 (2)	H15A—C15—H15B	107.8
C3—N1—C2	115.8 (3)	N1—C2—C1	125.0 (3)
C16—O4—C17	122.2 (2)	N1—C2—H2	117.5
C15—N5—C9	110.9 (2)	C1—C2—H2	117.5
C15—N5—C8	109.0 (2)	N1—C3—C4	124.4 (3)
C9—N5—C8	109.5 (2)	N1—C3—H3	117.8
N4—N3—N2	106.8 (2)	C4—C3—H3	117.8
N3—N4—C7	109.6 (2)	O2—C11—C12	110.5 (3)
C5—C4—C3	117.9 (3)	O2—C11—C13	101.8 (3)
C5—C4—H4	121	C12—C11—C13	110.8 (3)
C3—C4—H4	121	O2—C11—C14	109.4 (3)
O4—C17—C19	101.9 (3)	C12—C11—C14	112.6 (3)
O4—C17—C20	109.6 (3)	C13—C11—C14	111.1 (4)
C19—C17—C20	111.3 (3)	C11—C14—H14A	109.5
O4—C17—C18	109.4 (3)	C11—C14—H14B	109.5
C19—C17—C18	111.2 (3)	H14A—C14—H14B	109.5
C20—C17—C18	112.8 (3)	C11—C14—H14C	109.5
N2—C6—C7	105.3 (3)	H14A—C14—H14C	109.5
N2—C6—H6	127.4	H14B—C14—H14C	109.5
C7—C6—H6	127.4	C17—C19—H19A	109.5
N4—C7—C6	108.2 (3)	C17—C19—H19B	109.5
N4—C7—C8	121.8 (3)	H19A—C19—H19B	109.5
C6—C7—C8	130.0 (3)	C17—C19—H19C	109.5
O1—C10—O2	126.3 (3)	H19A—C19—H19C	109.5
O1—C10—C9	124.6 (3)	H19B—C19—H19C	109.5
O2—C10—C9	109.1 (2)	C2—C1—C5	117.5 (3)
N5—C9—C10	112.8 (2)	C2—C1—H1	121.2
N5—C9—H9A	109	C5—C1—H1	121.2
C10—C9—H9A	109	C11—C12—H12A	109.5
N5—C9—H9B	109	C11—C12—H12B	109.5
C10—C9—H9B	109	H12A—C12—H12B	109.5
H9A—C9—H9B	107.8	C11—C12—H12C	109.5
C6—N2—N3	110.1 (3)	H12A—C12—H12C	109.5
C6—N2—C5	129.3 (2)	H12B—C12—H12C	109.5
N3—N2—C5	120.5 (2)	C17—C18—H18A	109.5
C4—C5—C1	119.4 (3)	C17—C18—H18B	109.5
C4—C5—N2	120.3 (3)	H18A—C18—H18B	109.5
C1—C5—N2	120.3 (3)	C17—C18—H18C	109.5
N5—C8—C7	112.6 (2)	H18A—C18—H18C	109.5
N5—C8—H8A	109.1	H18B—C18—H18C	109.5
C7—C8—H8A	109.1	C17—C20—H20A	109.5
N5—C8—H8B	109.1	C17—C20—H20B	109.5
C7—C8—H8B	109.1	H20A—C20—H20B	109.5
H8A—C8—H8B	107.8	C17—C20—H20C	109.5
O3—C16—O4	125.4 (3)	H20A—C20—H20C	109.5
O3—C16—C15	125.8 (3)	H20B—C20—H20C	109.5
O4—C16—C15	108.8 (2)	C11—C13—H13A	109.5
N5—C15—C16	113.2 (2)	C11—C13—H13B	109.5
N5—C15—H15A	108.9	H13A—C13—H13B	109.5
C16—C15—H15A	108.9	C11—C13—H13C	109.5
N5—C15—H15B	108.9	H13A—C13—H13C	109.5
C16—C15—H15B	108.9	H13B—C13—H13C	109.5

### Hydrogen-bond geometry (Å, º)

Cg1 is the centroid of the N1/C1–C5 ring.

**Table:**

*D*—H···*A*	*D*—H	H···*A*	*D*···*A*	*D*—H···*A*
C2—H2···O1^i^	0.95	2.54	3.443 (4)	160
C6—H6···N1^ii^	0.95	2.31	3.252 (4)	173
C9—H9*B*···N3^iii^	0.99	2.50	3.449 (4)	160
C18—H18*A*···*Cg*1^iv^	0.98	2.91	3.864 (4)	166

Symmetry codes: (i) *x*, *y*+1, *z*; (ii) −*x*+2, *y*−1/2, −*z*+2; (iii) −*x*+1, *y*−1/2, −*z*+1; (iv) *x*, *y*−1, *z*.
